# Does HPV type affect outcome in oropharyngeal cancer?

**DOI:** 10.1186/1916-0216-42-9

**Published:** 2013-02-01

**Authors:** Anthony C Nichols, Sandeep S Dhaliwal, David A Palma, John Basmaji, Corina Chapeskie, Samuel Dowthwaite, Jason H Franklin, Kevin Fung, Keith Kwan, Brett Wehrli, Chris Howlett, Iram Siddiqui, Marina I Salvadori, Eric Winquist, Scott Ernst, Sara Kuruvilla, Nancy Read, Varagur Venkatesan, Biljana Todorovic, J Alex Hammond, James Koropatnick, Joe S Mymryk, John Yoo, John W Barrett

**Affiliations:** 1 Department of Otolaryngology, Head & Neck Surgery, The University of Western Ontario, Room B3-431A, 800 Commissioners Road East, London, N6A 5W9, , Ontario, Canada; 2 London Regional Cancer Program, London, Ontario, Canada; 3 Lawson Health Research Institute, London, Ontario, Canada; 4 Department of Oncology, The University of Western Ontario, London, Ontario, Canada; 5 Department of Pathology, The University of Western Ontario, London, Ontario, Canada; 6 Department of Paediatrics, Infectious Disease Division, The University of Western Ontario, London, Ontario, Canada; 7 Department of Microbiology and Immunology, The University of Western Ontario, London, Ontario, Canada

**Keywords:** Human papillomavirus, Oropharyngeal cancer, Epidemiology

## Abstract

**Background:**

An epidemic of human papillomavirus (HPV)-related oropharyngeal squamous cell cancer (OPSCC) has been reported worldwide largely due to oral infection with HPV type-16, which is responsible for approximately 90% of HPV-positive cases. The purpose of this study was to determine the rate of HPV-positive oropharyngeal cancer in Southwestern Ontario, Canada.

**Methods:**

A retrospective search identified ninety-five patients diagnosed with OPSCC. Pre-treatment biopsy specimens were tested for p16 expression using immunohistochemistry and for HPV-16, HPV-18 and other high-risk subtypes, including 31,33,35,39,45,51,52,56,58,59,67,68, by real-time qPCR.

**Results:**

Fifty-nine tumours (62%) were positive for p16 expression and fifty (53%) were positive for known high-risk HPV types. Of the latter, 45 tumors (90%) were identified as HPV-16 positive, and five tumors (10%) were positive for other high-risk HPV types (HPV-18 (2), HPV-67 (2), HPV-33 (1)). HPV status by qPCR and p16 expression were extremely tightly correlated (p < 0.001, Fishers exact test). Patients with HPV-positive tumors had improved 3-year overall (OS) and disease-free survival (DFS) compared to patients with HPV-negative tumors (90% vs 65%, p = 0.001; and 85% vs 49%, p = 0.005; respectively). HPV-16 related OPSCC presented with cervical metastases more frequently than other high-risk HPV types (p = 0.005) and poorer disease-free survival was observed, although this was not statistically significant.

**Conclusion:**

HPV-16 infection is responsible for a significant proportion of OPSCC in Southwestern Ontario. Other high-risk subtypes are responsible for a smaller subset of OPSCC that present less frequently with cervical metastases and may have a different prognosis.

## Introduction

The incidence of head and neck squamous cell carcinoma is generally decreasing [1,2], likely due to declining rates of smoking and alcohol use. An exception to this trend has been cancers affecting the oropharynx, which have increased by more than two-fold in some countries over the last three decades [3,4]. Infection by human papillomavirus (HPV) has been implicated as the cause of this dramatic rise in oropharyngeal squamous cell carcinomas (OPSCC), particularly those affecting the palatine and lingual tonsils [3]. Importantly, numerous studies have reported improved survival for patients with HPV-positive tumors [3,5,6]. Of the several high-risk strains of HPV identified, approximately 90 percent of HPV-positive OPSCC is attributed to type 16, while other types are rare [7].

There is a wide discrepancy in the fraction of OPSCC that are HPV-positive world-wide, with incidence rates ranging from 11% to as high as 93% [4,8]. In North America, most reports suggest that the incidence is approximately 60 percent [5,6]. These differences may be attributed to geographic population-based differences, as well as the varying sensitivity and specificity of different detection methods for the presence of HPV. We endeavoured to study patients from our catchment area in Southwestern Ontario, Canada to determine the incidence of HPV-related OPSCC and confirm whether HPV status conferred a survival advantage.

## Methods

### Study population

Study approval was obtained from the University of Western Ontario Research Ethics Board. A retrospective search of the London Regional Cancer Program (LRCP) database was performed to identify patients diagnosed with OPSCC from 2003 to 2009. Patient eligibility required: 1) a histologically confirmed diagnosis of squamous cell carcinoma, 2) no prior history of head and neck cancer, and 3) the availability of a pre-treatment primary site biopsy specimen for analysis. Patient data was extracted from a retrospective chart review, which included age at diagnosis, use of tobacco and alcohol, AJCC TNM stage, treatment regimen, and post-treatment follow-up information.

After the completion of cancer therapy, patients were followed closely at six to twelve week intervals by either a radiation oncologist or head and neck surgeon. Treatment response was evaluated by physical exam as well as computed tomography imaging as needed. Salvage neck dissection was undertaken within three months of the completion of radiation treatment if evidence of residual adenopathy was present radiographically or on physical exam. The presence of neck disease more than three months after diagnosis was considered a regional persistence.

### Deparaffinization

The formalin fixed paraffin embedded blocks from each patient’s primary site were sectioned (5 μm thick) and mounted on slides. The slides were then deparaffinized with 3 minute washes in xylene (100%) twice, followed by a 1:1 xylene:ethanol mix, then ethanol (100%) twice, followed by single washes in ethanol at 95%, 70% and 50%. Lastly, the slides were washed in water for 5 minutes.

### p16 immunohistochemistry

Citrate buffer was applied to the deparaffinized slides for heat induced epitope retrieval for 30 minutes, followed by treatment with 0.1% Triton X-100 in PBS. Endogenous biotin was quenched using an avidin/biotin kit (Biocare Medical, Concord CA,USA) and the endogenous peroxidase activity was blocked by H_2_0_2_ prior to primary antibody incubation. Specimens were incubated with a primary mouse monoclonal antibody against human p16 (MTM Laboratoris, Heidelberg, Germany) at 1:500 dilution. The Vectastain ABC kit, with a goat anti mouse secondary antibody, was used as per the manufacturer’s instructions. Antigen detection was done using diaminobenzidine chromogen (Biocare Medical, Concord, CA). Tissues were counterstained with Carazzi’s hematoxylin. Immunohistochemistry scoring was conducted by two head and neck pathologists (KK and BW) blinded to HPV status and patient outcome. Scoring was as described by Begum et al., with strong and diffuse staining (>80 percent of tumour cells) regarded as a positive result, and negative if absent or focal [9].

### DNA extraction

Deparaffinized tissue was scraped into a 1.5 ml eppendorf tube containing 50–100 μl (depending on the amount of tissue) of TE and proteinase K (final concentration 2 mg/ml) and incubated overnight at 65°C. Following proteinase K treatment, the samples were heated at 95°C for 10 minutes and allowed to cool to room temperature. 0.2 μl of each sample was used directly in the qPCR reactions.

### High-risk HPV detection by multiplex qPCR

In an effort to screen clinical samples for the presence of human papillomavirus (HPV), we designed a multiplex quantitative PCR to identify those samples that were HPV positive and to confirm the HPV type in the positive samples. We designed primer/probe sets (Table 1) against a 115 nucleotide (nt) fragment within exon 6 of GAPDH (internal control), a 110 nt region across E6-E7 of HPV-16, a 137 nt fragment across the HPV-18 E6-E7 region, and a 100 nt fragment across a region of E1 that had a high degree of homology to high-risk HPV types 16, 18, 31, 33, 35, 39, 45, 51, 52, 56, 58, 59, and 68. The multiplex PCR reactions were run on a Stratagene Mx3000P instrument using the conditions recommended in the Quantitech Multiplex handbook (Qiagen). Twenty μl reactions were heated for 15 minutes at 95°C to activate the amplification enzyme. This was followed by 40 cycles of 94°C for 60 seconds and 60°C for 90 seconds. Standard curves were produced from 10 fold serial dilutions of CaSki cell genomic DNA. Caski cell DNA was used as a positive control for HPV16 and HeLa cell genomic DNA was used as the HPV18 control. All samples were first tested with the type 16 and 18 primer/probe sets. Samples that were negative for types 16 and 18 were then tested with the broad-spectrum primer/probe set to detect the presence of other high-risk HPV types.

**Table 1:** PCR primers for HPV testing

**Name**	**Sequence 5′ to 3′**
GAPDH Forward	GCTCATTTGCAGGGGGGAGCC
GAPDH Reverse	CTGATGATCTTGAGGCTGTTG
GAPDH Probe	CY5-TCTGCCCCCTCTGCTGATGCCCCCATGTTCGTCATGGGA –BHQ2
HPV 16 Forward	TTGCAGATCATCAAGAACACGTAGA
HPV 16 Reverse	GTAGAGATCAGTTGTCTCTGGTTGC
HPV 16 Probe	JOE-AATCATGCATGGAGATACACCTACATTGCATGA –BHQ1
HPV 18 Forward	CAACCGAGCACGACAGGAACG
HPV 18 Reverse	TAGAAGGTCAACCGGAATTTTCAT
HPV 18 Probe	ROX-AATATTAAGTATGCATGGACCTAAGGCAACATTGCAA –BHQ2
HPV all Forward (E1)	CCTATAGTACATTTAAAAGGTG
HPV all Reverse (E1)	CNTGTCCAATGCCAGGTAGATG
HPV all Probe (E1)	FAM-AATAGTTTAAAATGTTTAAGATATAG-BHQ1
HPV other Forward (E2)	GCATTATATTGGTATAGAACAGG
HPV other Reverse (E2)	TCATTRTCASATGCCCATTGYACC

*HPV* human papillomavirus; *R* puRine (A or T); *S* Strong (C or G); *Y* pYrimidine (C or T).

### High-risk HPV detection by conventional PCR

Tumors that tested positive for HPV infection by qPCR with the broad spectrum primer/probe set designed against the E2 region of HPV were tested with a second broad spectrum set of primers against the E1 region of HPV by conventional PCR (Table 1). PCR reactions (20 μl) were prepared using 0.2 μl of patient template DNA and amplified with Phusion DNA polymerase (Fisher Scientific, Toronto, Canada) following a hotstart (98°C for 30 sec followed by 37 cycles of 98°C for 10 sec, 55°C for 10 sec, 72°C for 30 sec). Amplified products were resolved on 1% agarose gels. Bands of interest were extracted from agarose gel slices, purified on columns (Bio Basic, Markham, Canada) and sequenced with the appropriate primer. Results were used to query the Genbank Database to identify the corresponding HPV type.

### Statistical analysis

Statistical analysis was performed using SPSS with the exact tests package (IBM, Armonk, NY). For all analyses, a *p* value of 0.05 or less was considered statistically significant. Patient variables were compared using the Fisher exact test. Disease-free survival (DFS) was defined as the time of completion of treatment to local, regional, or distant recurrence. Survival curves were created using the method of Kaplan and Meier. The Cox proportional hazard model was used to estimate the relative hazard of mortality or recurrence over the follow-up period and to perform univariate and multivariate analysis. Multivariate analysis began with a fit to all patient variables, followed by backward stepwise regression using the Akaike information criterion (stepAIC function) and retention of variables in the final model having *p* values of 0.05 or less.

## Results

### HPV-16 presents more frequently with neck metastases than other high-risk HPV types

A total of 95 patients with OPSCC were identified. The majority of patients were treated with organ preservation strategies (86/95, 90.5%) as opposed to primary surgery (9/95, 9.5%) (Table 2). Patients only underwent primary surgery if they refused primary radiation (2), had gross bony involvement on imaging (5) or had medical contraindications to radiation (2, severe scleroderma). Fifty-nine patients had tumour biopsy specimens that were p16 positive. Real-time qPCR for high-risk HPV types clearly separated HPV-positive tumors (all had a cycle threshold C_T_ < 30) and negative tumors (C_T_ > 35). Overall, fifty tumors were positive for HPV, including 45 positive for HPV-16 (90%) and 2 positive for HPV-18. The broad-spectrum primer/probe set detected three other HPV-positive tumors, which were determined to be type 33 (1 tumor) and type 67 (2 tumors) by sequencing of PCR products. The identification of HPV-67 was surprising because the high-risk HPV-67 sequence was not included in our efforts to design broad-spectrum primers. Regardless, sequencing of the 150 nt product was highly consistent with the published HPV-67 sequence by BLAST analysis [10] (p < 10^-39^, accession D21208). p16-positivity was tightly correlated with HPV status (*p* < 0.0001, Fishers exact test), further supporting its use as a surrogate marker of HPV infection. Analysis of patient characteristics and HPV-positive OPSCC revealed associations with age <60, tonsil or base of tongue primary site, and minimal smoking history (*p* < 0.05, Table 2). Patients with HPV-16-positive tumors were compared to those with tumors positive for other high-risk HPV types. HPV-16-positive tumors were significantly more likely to have neck metastases (43/45 versus 2/5, *p* = 0.005, Table 3). One of two HPV-18-positive patients and one of two HPV-67-positive patients had lymph node involvement. The sole HPV-33-positive patient was node negative.

**Table 2:** Correlation of HPV status with patient and tumor factors

			**All high-risk HPV**			**HPV-16 only**		
**Patient characteristic**		**Total**	**Negative**	**Positive**	p*	**Negative**	**Positive**	p*
**Age**	<60	51	16	35	**<0.001**	18	33	**<0.001**
	> = 60	44	29	15		32	12	
**Gender**	Male	75	32	43	0.08	37	38	0.32
	Female	20	13	7		13	7	
**Subsite**	Tonsil		21	31	**0.02**	23	29	**0.01**
	Base of Tongue		10	15		12	13	
	Other		14	4		15	3	
**T Classification**	1	14	4	10	0.27	4	10	0.19
	2	25	11	14		14	11	
	3	30	18	12		19	11	
	4	26	12	14		13	13	
**N Classification**	0	18	13	5	0.09	16	2	**0.002**
	1	13	7	6		8	5	
	2	54	21	33		22	32	
	3	10	4	6		4	6	
**Overal Stage**	1	2	1	1	0.49	1	1	**0.02**
	2	9	5	4		8	1	
	3	13	8	5		10	4	
	4	71	31	40		31	39	
**Smoking**	never smokers	20	4	16	**0.006**	5	1	**0.001**
	1-9 py	6	2	4		2	4	
	10-19 py	6	1	5		1	5	
	>20 py	57	35	22		39	18	
	Unknown	6	3	3		3	3	
**Alcohol**	< 21 drinks	70	30	40	0.37	34	36	0.45
**(drinks/wk)**	>21 drinks	17	10	7		11	6	
	Unknown	8	5	3		5	3	
**Treatment**	CRT	62	29	33	0.78	30	32	0.55
	Radiation	11	5	6		8	3	
	Induction + CRT	13	5	8		6	7	
	Surgery + CRT	5	3	2		3	2	
	Surgery + RT	2	1	1		1	1	
	Surgery	2	2	0		2	0	
**HPV Type**	16	45	0	45		0	45	
	18	2	0	2		2	0	
	33	1	0	1		1	0	
	67	2	0	2		2	0	
**p16**	Negative	35	34	1	**<0.001**	34	1	**<0.001**
	Positive	59	10	49		15	44	

*HPV* human papillomavirus, *py* pack years, *RT* radiation, *CRT* chemoradiation.

* Fisher exact test.

**Table 3:** Comparison of HPV-16 and other high-risk (non-16) HPV types

			**HPV-Positive**		
		**Total**	**High-risk HPV (non-16)**	**HPV16**	p*
**Age**	<60	35	2	33	0.15
	> = 60	15	3	12	
**Gender**	Male	43	5	38	1
	Female	7	0	7	
**Subsite**	Tonsil	31	2	29	0.33
	Base of Tongue	15	2	13	
	Other	4	1	3	
**T Classification**	1	10	0	10	0.58
	2	14	3	11	
	3	12	1	11	
	4	14	1	13	
**N Classification**	0	5	3	2	**0.005**
	1-3	45	2	43	
**Overal Stage**	1	1	0	1	**<0.001**
	2	4	3	1	
	3	5	1	4	
	4	40	1	39	
**Smoking**	never smokers	16	1	15	0.73
	1-9 py	4	0	4	
	10-19 py	5	0	5	
	>20 py	22	4	18	
	Unknown	3	0	3	
**Alcohol**	< 21 drinks	40	4	36	0.69
**(drinks/wk)**	>21 drinks	7	1	6	
	Unknown	3	0	3	
**Treatment**	CRT	33	1	32	**0.02**
	Radiation	6	3	3	
	Induction + CRT	8	1	7	
	Surgery + CRT	2	0	2	
	Surgery + RT	1	0	1	
	Surgery	0	0	0	
**p16**	Negative	1	0	1	1
	Positive	49	5	44	

*HPV* human papillomavirus, *py* pack years, *RT* radiation, *CRT* chemoradiation.

* Fisher exact test.

### HPV-positive patients experience improved survival

**Figure 1:**
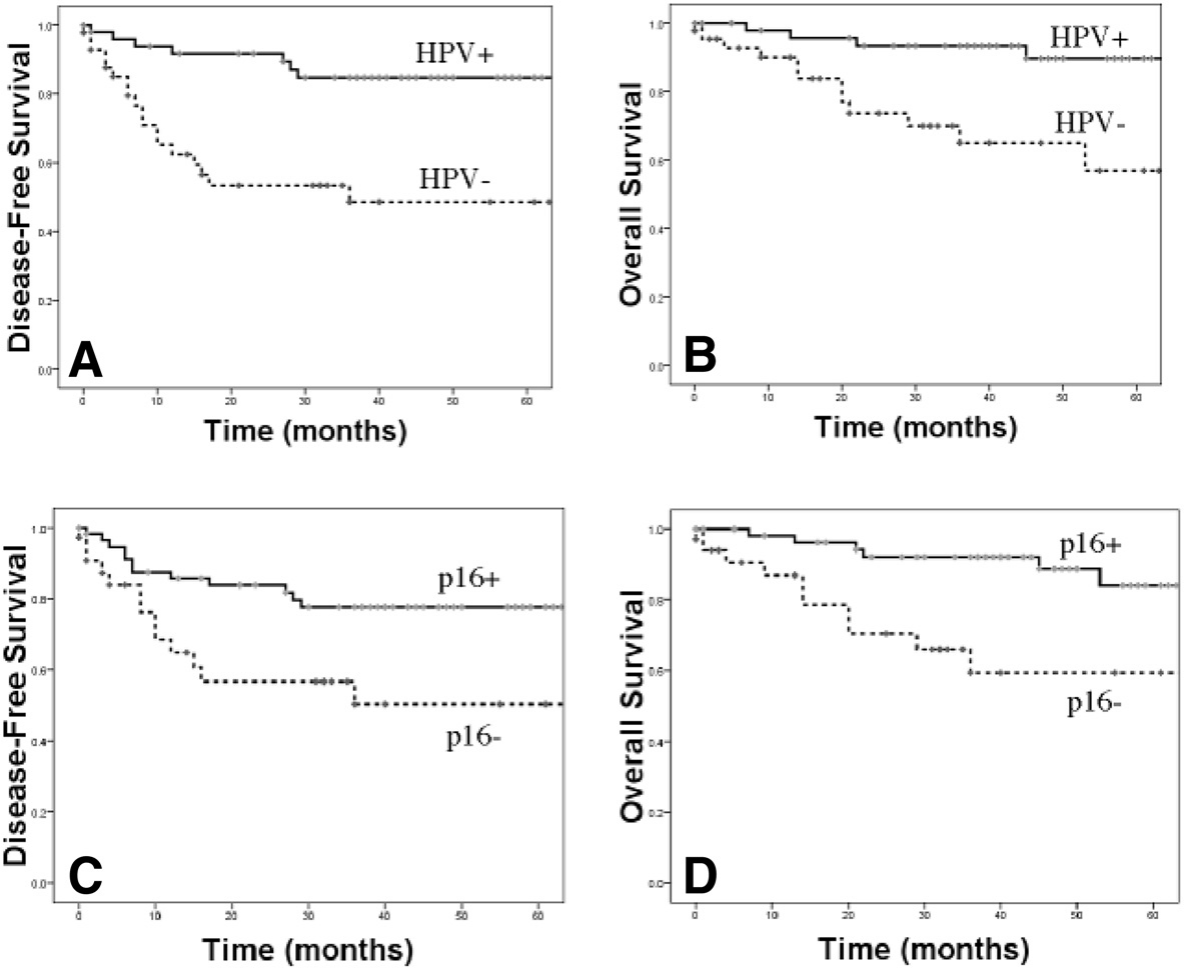
Disease-free and overall survival by HPV status (A and B) and p16 status (C and D).

Median follow-up time for the study was 39 months (range: 1 to 91 months). A total of 26 of 95 patients (27%) recurred, and eleven (42%) of these patients died of disease. Five patients died of other causes. The three-year disease-free (DFS) and overall survival (OS) for all patients was 75 % and 85%, respectively. Univariate analysis identified age, p16 status and the presence of any high-risk HPV type as predictors of disease-free and overall survival (Table 4 and Figure 1A and 1B). Three year DFS and OSP rates for patients with HPV-positive versus HPV-negative tumors were 85% vs 49%, and 90% vs 65%; respectively. p16 positivity was also associated with improved DFS and OS (p < 0.05, Table 4 and Figure 1C and 1D). Multivariate analysis revealed high-risk HPV infection as the only independent predictor of disease-free and overall survival (p = 0.001 and p = 0.004, respectively, Table 4). As multiple treatments were used, we repeated the survival analysis using only the data from the sixty-two patients that received platinum-based concurrent chemoradiation to remove this potential confounding factor. High-risk HPV infection once again was the only predictor of overall and disease-free survival (p < 0.005, data not shown).

**Table 4:** Univariate and multivariate relations to disease free survival

		**DFS (Univariate analysis)**		**DFS (Multivariate analysis)**	
		HR (95% CI)*	P**	HR (95% CI)*	P**
Age	> = 60 vs <60	3.34 (1.44-7.71)	**0.01**		
Sex	Male vs Female	1.06 (0.40-2.81)	0.91		
Site	Tonsil, BOT vs Other				
T stage	3,4 vs 1,2	1.00 (0.46-2.17)	0.99		
N stage	N + vs N0	0.51 (0.22-1.18)	0.11		
Smoking	>10 py vs < 10 py or unknown	1.54 (0.64-3.72)	0.34		
Alcohol	>21 drinks vs <21 drinks and Unknown	0.94 (0.33-2.75)	0.92		
p16	Positive vs Negative	0.41 (0.19-0.90)	**0.025**		
All High-Risk HPV	Positive vs Negative	0.25 (0.11-0.58)	**0.001**	0.24 (0.10-0.56)	**0.001**
HPV 16 only	Positive vs Negative	0.27 (0.11-0.65)	**0.003**		
		**OS (Univariate Analysis)**		**OS (Multivariate Analysis)**	
		HR (95% CI)*	P**	HR (95% CI)*	P**
Age	> = 60 vs <60	3.05 (1.06-8.80)	**0.04**		
Sex	Male vs Female	0.70 (0.23-2.17)	0.53		
Site	Tonsil, BOT vs Other	1.38 (0.39-4.90)	0.61		
T stage	3,4 vs 1,2	1.76 (0.61-5.07)	0.30		
N stage	N + vs N0	0.37 (0.13-1.01)	0.05		
Smoking	>10 py vs < 10 py or unknown	2.34 (0.67-8.25)	0.19		
Alcohol	>21 drinks vs <21 drinks and Unknown	1.16 (0.33-4.09)	0.81		
p16	Positive vs Negative	0.24 (0.09-0.67)	**0.006**		
All High-Risk HPV	Positive vs Negative	0.20 (0.06-0.62)	**0.005**	0.19 (0.06-0.60)	**0.004**
HPV 16 only	Positive vs Negative	0.17 (0.05-0.59)	**0.006**		

*DFS* disease-free survival, *OS* overal survival, *HR* hazard ratio, *CI* confidence interval, *py* pack years, *HPV* human papillomavirus.

*CI (confidence interval) by Cox proportional hazard analysis.

**Wald test.

**Figure 2:**
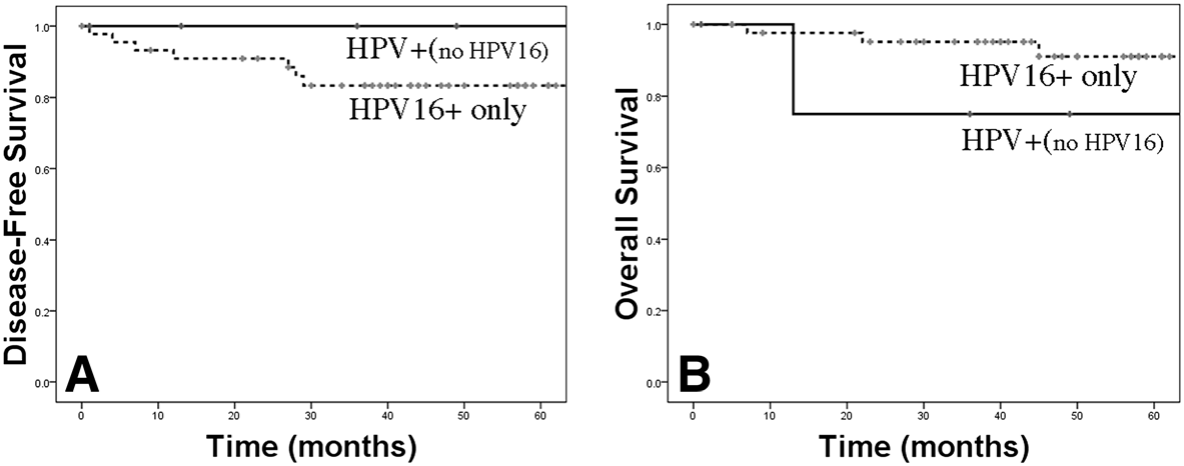
Disease-free (A) and overall survival (B) for HPV-positive patients stratified by HPV type.

Seven of 45 HPV-16-positive patients recurred, while none of the five patients with tumors due to non-16 high-risk HPV types developed a recurrence (DFS 83.3% vs 100%, p = 0.94, Figure 2A). No difference in overall survival between the HPV-16 and the HPV-other high-risk group was detected (Figure 2B). The only death in the patients with non-16 HPV-positive tumors was due to a myocardial infarction in an 87-year-old patient 13 months after diagnosis. Together, these data confirm the significance of HPV as a prognostic factor in OPSCC, and suggests that stage at presentation may differ between high-risk HPV types.

## Discussion

Recent studies have clearly described the current epidemic of HPV-positive oropharyngeal squamous cell carcinoma (OPSCC) in North America and beyond [3,4]. Using tumors from patients from three American population based cancer registries in the Surveillance, Epidemiology and End Results (SEER) program, Chaturvedi et al [3] conclusively demonstrated that HPV-positive OPSCC has increased markedly (225%) over the last three decades. Improved survival for patients with HPV-positive OPSCC was reported and our results agree with this finding. Patients tend to be younger, non-smokers, and non-drinkers with low rates of comorbid illness [3,5,6]. The observed high cure rates in this high functioning cohort of younger patients has led to an increased emphasis on post-treatment quality of life, as treatment side effects will potentially need to be endured for decades. Consequently, several studies have been initiated with the goal of the long-term sequelae of therapy for patients with HPV-positive OPSCC, such as the substitution of cetuximab for cisplatin in RTOG-1016.

However, a fraction of these patients still relapse and succumb to disease following conventional therapy. Ang et al [5] demonstrated that 21.2% of patients with HPV-positive tumors relapsed within 3 years, with 13.6% of these cases due to locoregional recurrence, and 8.7% due to the development of distant metastases. The biological reasons for this difference in treatment effectiveness are poorly understood. A partial explanation may be the specific HPV type implicated. In cervical cancer, HPV types 18 and 58 are associated with a poorer prognosis, while HPV-31 portends improved survival compared with HPV-16 [11-13]. To date only one study has demonstrated investigated a differential response or clinical presentation by HPV type in head and neck cancer. Rautava et al., retrospectively analyzed 106 patients with tumors from multiple head and neck sites including 31 oropharyngeal patients [14]. HPV genotyping was performed with a Luminex based Multimetrix® genotyping kit. In contrast with most studies, they found a very high rate of infection in non-oropharyngeal sites including the oral cavity (76%) and larynx (40%), as well as very high rate of co-infection (44%) and the presence of low-risk subtypes 6 and 11 (20%) [2,15]. HPV was not found to be predictive of survival and there was no difference in outcome based on HPV type [14].

Although limited conclusions can be drawn given the small numbers of HPV types other than HPV-16 in our cohort, our data suggests that HPV-16 cancers may present more frequently with lymph node metastases and may have a poorer outcome compared with non-16 high-risk HPV types. Given the low frequency of HPV-positive OPSCC presentations that are not due to HPV-16, a very large multi-institutional series with HPV genotyping would need to be assembled in order to answer this question definitively.

A tremendous step forward in the battle against the multiple cancers caused by HPV is the implementation of HPV vaccination programs worldwide. The quadrivalent HPV vaccine is highly effective in preventing benign and precancerous lesion lesions due to HPV types 6, 11, 16 and 18 including cervical and anal dysplasia [16,17]. Due to the significant lag time between infection and the development of malignancy, it will take decades to determine if these vaccines are effective in preventing anogenital and head and neck cancers. Indeed, most individuals develop HPV infection in their teens and twenties [18], however the average age of diagnosis of HPV-positive patients in our study was 58 years (range, 43–86). Due to this significant time gap as well as low rates of use of this vaccine in both males and females, the burden of HPV-related cancers will be an important clinical problem for the foreseeable future.

HPV-positive cancers are fundamentally distinct from traditional tobacco and alcohol related cancers [2]. Viral oncogenes E6 and E7 cause the degradation of wild-type p53 and pRb, respectively, while p16 is overexpressed. This is in contrast to frequent mutations of p53 and loss of p16 seen in conventional head and neck cancer [2]. The differences between HPV-positive and conventional head and neck cancers was further highlighted in two landmark papers by Stransky and Agrawal that reported large-scale whole exome sequencing of head and neck cancers [19,20]. These studies revealed approximately a two to four-fold increase in number of mutations in HPV-negative compared with HPV-positive cancers and confirmed the differences in p53 and p16 outlined above. Despite these differences, there were significant overlaps in the mutational profiles of the two cancer types, including mutations in the NOTCH receptors (NOTCH1-3), HRAS, and PIK3CA that were seen in both HPV-positive and negative disease. Potentially, high-throughput technologies including expression microarrays, RNA-seq, and exome sequencing of large cohorts of HPV-positive OPSCC can identify biomarkers of local, regional and distant relapse as well as biological differences between cancers due to different HPV types. Patients at high-risk of failure could undergo treatment intensification with the goal of increasing survival, while low-risk patients could have their treatment de-intensified in order to optimize post-treatment quality of life. This would be a significant step towards customized care for patients with HPV-positive HNSCC.

## Conclusions

HPV infection is the etiologic factor for a significant proportion of OPSCC in Southwestern Ontario as has been observed worldwide. The majority of cases are attributable to HPV-16, while other high-risk subtypes are responsible for the remainder. HPV-16-positive OPSCC may present at a more advanced stage than those due to other high-risk HPV types. Larger studies are required to confirm this observation and determine if different HPV types influence prognosis.

## Competing interest

This study was supported by an investigator initiated grant from Merck Canada, Inc. The authors have no other disclosures.

## Authors’ contribution

This project has been a coordinated effort designed by the London Health Sciences Centre Head and Neck multidisciplinary team. Specifically, medical oncologists (EW, SK, SE), radiation oncologists (DP, NR, VV, JAH), head and neck surgeons (AN, KF, JY, JF, SD) and translational scientists (AN, JSM, JK, JWB) planned this study together from its initiation. Dr. Marina Salvadori (MI), an HPV and vaccine expert, was also involved with the study design. Pathologists (BW and KK) were responsible for screening all specimens for the presence of tumor. As this study involved a large retrospective chart review, specimen retrieval, DNA extraction, and HPV testing multiple trainees were involved. Specifically, patient data was extracted by SSD, CC, JB, and DNA was extracted by AN and JWB. All HPV testing was done by the senior author JWB. The manuscript was written by AN and JWB and critically revised by all authors. All authors read and approved the final manuscript.

## Funding

This study was supported by a research grant from Merck Canada Inc.

This material has never been published and is not currently under evaluation in any other peer-reviewed publication.

## References

[B1] SEER Cancder Statistics Review, 1975–2008 2011 Bethesda, MD: National Cancer Institute http://seer.cancer.gov/csr/1975_2008/

[B2] Marur S D'Souza G Westra WH Forastiere AA HPV-associated head and neck cancer: a virus-related cancer epidemic Lancet Oncol 2010 11 8 781 9 10.1016/S1470-2045(10)70017-6 20451455 PMC5242182

[B3] Chaturvedi AK Engels EA Pfeiffer RM Human papillomavirus and rising oropharyngeal cancer incidence in the United States J Clin Oncol 2011 29 32 4294 301 10.1200/JCO.2011.36.4596 21969503 PMC3221528

[B4] Nasman A Attner P Hammarstedt L Incidence of human papillomavirus (HPV) positive tonsillar carcinoma in Stockholm, Sweden: an epidemic of viral-induced carcinoma? Int J Cancer 2009 125 2 362 6 10.1002/ijc.24339 19330833

[B5] Ang KK Harris J Wheeler R Human papillomavirus and survival of patients with oropharyngeal cancer N Engl J Med 2010 363 1 24 35 10.1056/NEJMoa0912217 20530316 PMC2943767

[B6] Nichols AC Faquin WC Westra WH HPV-16 infection predicts treatment outcome in oropharyngeal squamous cell carcinoma Otolaryngol Head Neck Surg 2009 140 2 228 34 10.1016/j.otohns.2008.11.025 19201294

[B7] Kreimer AR Clifford GM Boyle P Franceschi S Human papillomavirus types in head and neck squamous cell carcinomas worldwide: a systematic review Cancer Epidemiol Biomarkers Prev 2005 14 2 467 75 10.1158/1055-9965.EPI-04-0551 15734974

[B8] Szkaradkiewicz A Kruk-Zagajewska A Wal M Jopek A Wierzbicka M Kuch A Epstein-Barr virus and human papillomavirus infections and oropharyngeal squamous cell carcinomas Clin Exp Med 2002 2 3 137 41 10.1007/s102380200019 12447611

[B9] Begum S Gillison ML Ansari-Lari MA Shah K Westra WH Detection of human papillomavirus in cervical lymph nodes: a highly effective strategy for localizing site of tumor origin Clin Cancer Res 2003 9 17 6469 75 14695150

[B10] Johnson M Zaretskaya I Raytselis Y Merezhuk Y McGinnis S Madden TL NCBI BLAST: a better web interface Nucleic Acids Res 2008 36 Web Server issue W5 9 18440982 10.1093/nar/gkn201 PMC2447716

[B11] Burger RA Monk BJ Kurosaki T Human papillomavirus type 18: association with poor prognosis in early stage cervical cancer J Natl Cancer Inst 1996 88 19 1361 8 10.1093/jnci/88.19.1361 8827013

[B12] Huang LW Chao SL Hwang JL Human papillomavirus-31-related types predict better survival in cervical carcinoma Cancer 2004 100 2 327 34 10.1002/cncr.20003 14716768

[B13] Wright JD Li J Gerhard DS Human papillomavirus type and tobacco use as predictors of survival in early stage cervical carcinoma Gynecol Oncol 2005 98 1 84 91 10.1016/j.ygyno.2005.03.038 15894364

[B14] Rautava J Kuuskoski J Syrjanen K Grenman R Syrjanen S HPV genotypes and their prognostic significance in head and neck squamous cell carcinomas J Clin Virol 2012 53 2 116 20 10.1016/j.jcv.2011.11.005 22177275

[B15] Fakhry C Westra WH Li S Improved survival of patients with human papillomavirus-positive head and neck squamous cell carcinoma in a prospective clinical trial J Natl Cancer Inst 2008 100 4 261 9 10.1093/jnci/djn011 18270337

[B16] Munoz N Manalastas R Jr Pitisuttithum P Safety, immunogenicity, and efficacy of quadrivalent human papillomavirus (types 6, 11, 16, 18) recombinant vaccine in women aged 24–45 years: a randomised, double-blind trial Lancet 2009 373 9679 1949 57 10.1016/S0140-6736(09)60691-7 19493565

[B17] Palefsky JM Giuliano AR Goldstone S HPV vaccine against anal HPV infection and anal intraepithelial neoplasia N Engl J Med 2011 365 17 1576 85 10.1056/NEJMoa1010971 22029979

[B18] Kliewer EV Demers AA Elliott L Lotocki R Butler JR Brisson M Twenty-year trends in the incidence and prevalence of diagnosed anogenital warts in Canada Sex Transm Dis 2009 36 6 380 6 10.1097/OLQ.0b013e318198de8c 19556932

[B19] Agrawal N Frederick MJ Pickering CR Exome sequencing of head and neck squamous cell carcinoma reveals inactivating mutations in NOTCH1 Science 2011 333 6046 1154 7 10.1126/science.1206923 21798897 PMC3162986

[B20] Stransky N Egloff AM Tward AD The mutational landscape of head and neck squamous cell carcinoma Science 2011 333 6046 1157 60 10.1126/science.1208130 21798893 PMC3415217

